# Prevalence of Dermatologic Side Effects of Mood Stabilizers in Bipolar Disorder: A Systematic Review and A Meta-Analysis

**DOI:** 10.1192/j.eurpsy.2025.586

**Published:** 2025-08-26

**Authors:** F. Pampaloni, M. Ercis, D. M. R. Davis, M. Starace, B. M. Piraccini, A. Ozerdem, B. Singh, M. A. Frye, J. Blacker, M. Veldic

**Affiliations:** 1 Psychiatry and Psychology, Mayo Clinic, Rochester, United States; 2 Unit of Dermatology, IRCCS University Hospital of Bologna, Bologna, Italy; 3 Department of Dermatology, Mayo Clinic, Rochester; 4 Psychiatry and Behavioral Sciences, University of Minnesota, Minneapolis, United States

## Abstract

**Introduction:**

Bipolar disorder (BD) is a chronic illness affecting approximately 2-3% adults worldwide. Mood stabilizers, such as lithium, valproate, carbamazepine, and lamotrigine are mainstays of the treatment. Despite their benefits, mood stabilizers carry a risk of side effects, which can lead to treatment discontinuation and non-adherence rates ranging from 10 to 60% in BD patients (Dols *et al.* Int Clin Psychopharmacol 2013; 28, 287-296). Dermatologic side effects, also known as cutaneous adverse drug reactions, are particularly distressing, often impacting patients’ self-esteem and social interactions, and contributing to non-compliance. These reactions can range in severity from mild rashes, acneiform eruptions, hair loss and psoriasis to severe, life-threatening conditions like Stevens-Johnson syndrome (SJS) and toxic epidermal necrolysis (TEN) (Mitkov *et al.* Psychosomatics 2014; 55, 1-20).

**Objectives:**

Despite the well-documented association between mood stabilizers and dermatologic adverse effects (AE), the overall prevalence of these reactions across mood stabilizers remains unclear. This systematic review and meta-analysis aims (1) to estimate the prevalence of dermatologic AE associated with mood stabilizer (lithium, valproate, carbamazepine and lamotrigine) use in patients with BD, and (2) to summarize the available evidence on the onset and timing of these reactions.

**Methods:**

We searched Ovid MEDLINE®, Embase, Cochrane Library, Web of Science, Scopus, and PsycINFO from 1970 onward for studies on dermatologic AEs in BD patients treated with lithium, valproate, carbamazepine, or lamotrigine (CRD42022357268). Study selection, data extraction, and bias risk assessment were performed by two reviewers. Meta-analyses were conducted to estimate the prevalence rates for dermatologic AEs.

**Results:**

The initial database searches yielded 5,354 studies. 47 articles were deemed relevant and included in this systematic review. Study designs included 16 randomized controlled trials, 10 non-randomized open-label trials, 12 cohort studies, and 9 cross-sectional studies.

Lithium was associated with acneiform eruptions in 4.4% (95% CI: 1.0-17.0%), rash in 1.8% (95% CI: 0.7-4.8%), and hair loss in 1.7% (95% CI: 0.4%-6.4%) of patients. For valproate, hair loss was observed in 4.6% of patients (95% CI: 3.0-6.7%) and rash in 2.9% (95% CI: 1.6-5.3%). Carbamazepine was associated with rash in 6.0% of patients (95% CI: 4.4-7.6%), but severe reactions such as SJS and TEN were not reported. Lamotrigine had the highest rash prevalence with 9.2% (95% CI: 7.2-11.8%), while severe reactions were rare (0.04%, 95% CI: 0.00-0.63%).

**Conclusions:**

Mood stabilizers showed varying levels of dermatologic AEs, but severe reactions were rare. Future studies should explore factors influencing these outcomes, their impact on quality of life and treatment participation, and potential management strategies.

**Disclosure of Interest:**

None Declared

